# Sensing of Fatty Acids for Octanoylation of Ghrelin Involves a Gustatory G-Protein

**DOI:** 10.1371/journal.pone.0040168

**Published:** 2012-06-29

**Authors:** Sara Janssen, Jorien Laermans, Hiroshi Iwakura, Jan Tack, Inge Depoortere

**Affiliations:** 1 Translational Research Center for Gastrointestinal Disorders, Catholic University of Leuven, Leuven, Belgium; 2 Departments of Medicine, Clinical Science, Endocrinology, and Metabolism, Translational Research Center, Kyoto University Graduate School of Medicine, Kyoto University Hospital, Kyoto, Japan; University of Cordoba, Spain

## Abstract

**Background:**

Ghrelin is an important regulator of energy – and glucose homeostasis. The octanoylation at Ser^3^ is essential for ghrelin’s biological effects but the mechanisms involved in the octanoylation are unknown. We investigated whether the gustatory G-protein, α-gustducin, and the free fatty acid receptors GPR40 and GPR120 are involved in the fatty acid sensing mechanisms of the ghrelin cell.

**Methods:**

Wild-type (WT) and α-gustducin knockout (gust^−/−^) mice were fed a glyceryl trioctanoate-enriched diet (OD) during 2 weeks. Ghrelin levels and gastric emptying were determined. Co-localization between GPR40, GPR120 and ghrelin or α-gustducin/α-transducin was investigated by immunofluorescence staining. The role of GPR120 in the effect of medium and long chain fatty acids on the release of ghrelin was studied in the ghrelinoma cell line, MGN3-1. The effect of the GPR40 agonist, MEDICA16, and the GPR120 agonist, grifolic acid, on ghrelin release was studied both *in vitro* and *in vivo.*

**Results:**

Feeding an OD specifically increased octanoyl ghrelin levels in the stomach of WT mice but not of gust^−/−^ mice. Gastric emptying was accelerated in WT but not in gust^−/−^ mice. GPR40 was colocalized with desoctanoyl but not with octanoyl ghrelin, α-gustducin or α-transducin positive cells in the stomach. GPR120 only colocalized with ghrelin in the duodenum. Addition of octanoic acid or α-linolenic acid to MGN3-1 cells increased and decreased octanoyl ghrelin levels, respectively. Both effects could not be blocked by GPR120 siRNA. MEDICA16 and grifolic acid did not affect ghrelin secretion *in vitro* but oral administration of grifolic acid increased plasma ghrelin levels.

**Conclusion:**

This study provides the first evidence that α-gustducin is involved in the octanoylation of ghrelin and shows that the ghrelin cell can sense long- and medium-chain fatty acids directly. GPR120 but not GPR40 may play a role in the lipid sensing cascade of the ghrelin cell.

## Introduction

Ghrelin, a 28 amino-acid peptide, is synthesized in the X/A-like endocrine cells of the gastric mucosa [1]. Besides its potent stimulatory effect on growth hormone secretion, ghrelin also plays a prominent role in the regulation of energy – and glucose homeostasis [2], [3]. Ghrelin regulates short-term energy homeostasis by increasing hunger and food intake, an effect that is mediated by the activation of neuropeptide Y and agouti-related peptide producing neurons in the hypothalamus [4], [5]. In addition, ghrelin is also implicated in the regulation of long-term energy balance by promoting weight gain and adiposity [6], [7], [8].

The meal-related fluctuations in plasma ghrelin levels indicate that ghrelin is a physiological meal initiator [9]. While the preprandial increase in ghrelin levels is produced by norepinephrine released from sympathetic neurons acting directly on β_1_ receptors at the ghrelin cell [10], the postprandial ghrelin suppression is dependent on the caloric value and macronutrient composition of the meal [11], [12], [13]. Lipids are less effective at suppressing ghrelin levels than proteins, which in turn are less potent than carbohydrates. The nutrient sensing mechanisms of the ghrelin cell that determine these effects are so far unknown.

Ghrelin appears mainly in two forms, desoctanoyl ghrelin which is the dominant form in the plasma and octanoyl ghrelin [14]. Octanoylated ghrelin is produced post-translationally by modification of Ser^3^ with an eight carbon-fatty acid, octanoate, which is essential to bind and activate the ghrelin receptor. This octanoylation takes place in the lumen of the endoplasmatic reticulum of the ghrelin cell and is mediated by a membrane-bound O-acyl transferase known as ghrelin O-acyltransferase (GOAT) [15], [16].

Even before the discovery of GOAT it has been reported that ingested medium chain fatty acids (MCFA) and medium-chain triglycerides serve as a direct source of fatty acids in the acyl modification of ghrelin [17]. The mechanisms involved in fatty acid sensing of the ghrelin cells are not revealed yet, but we hypothesize that this may involve free fatty acid receptors (FFAR). GPR40 and GPR120 are G-protein coupled receptors whose endogenous ligands are medium and long chain fatty acids (LCFA) [18], [19]. GPR40 is expressed in the brain and gastrointestinal tract but mainly in pancreatic β-cells where the receptor mediates free fatty acid (FFA)-stimulated insulin secretion [20]. GPR120 is abundantly expressed in the distal intestine, and functions mainly as a receptor for unsaturated LCFA such as α-linolenic acid [21]. The stimulation of GPR120 by FFAs promotes the secretion of glucagon-like peptide-1 (GLP-1) and cholecystokinin (CCK) from the enteroendocrine cell line STC-1 [21], [22]. GPR40 is involved in the secretion of CCK from native I cells in response to dietary fat [23]. However, the downstream signaling pathway of these FFAR remains unclear.

Approximately 40% of the GPR120-positive cells in the taste buds express α-gustducin [24]. This gustatory G-protein, together with α-transducin, is a key downstream transduction component of the sweet, bitter and umami taste receptors [25], [26]. We recently showed that α-gustducin also plays a role in the effect of bitter agonists on ghrelin secretion [27]. Both α-gustducin and α-transducin are colocalized with octanoyl containing ghrelin cells in the mouse stomach [27]. In addition α-gustducin but not α-transducin is also present in the brush cells in close contact with some ghrelin cells. These brush cells may function as input cells to convey signals from the lumen via PGP9.5-innervating fibers to neighboring cells [28]. α-gustducin is also present in the mouse small intestine where it is colocalized with 5-HT and GLP-1 [29]. Also in humans expression of α-gustducin has been demonstrated in stomach and small intestine [30], [31].

The aim of the present study was to investigate whether the free fatty acid receptors, GPR40 or GPR120 and/or α-gustducin are involved 1) in the octanoylation process of ghrelin by medium chain fatty acids 2) in the effect of long chain fatty acids on the release of ghrelin.

## Material and Methods

### Animals

Male C57BL/6 WT mice were obtained from Janvier (Le Genest St. Isle, France), gust^−/−^ mice were kindly provided by Dr. Margolskee (Monell Chemical Senses center, Philadelphia) and ghrelin receptor knockout (GHS-R^−/−^) mice were developed as previously described and bred in our animal facility [32]. All mice were between 10 and 14 weeks of age, housed in a temperature-controlled environment (20–22°C) under a 14-h:10-h light-dark cycle and had *ad libitum* access to food and drinking water. All experimental procedures were approved by the Ethical committee for Animal Experiments of the Catholic University of Leuven.

### Experimental Design: Octanoyl Modification of Ghrelin

Both WT, gust^−/−^ or GHS-R^−/−^ mice were fed a control diet (2016 Teklad Global 16% Protein Rodent Diet, Harlan Laboratories) or a control diet enriched (5%) with glyceryl trioctanoate. Food intake and body weight were measured daily and gastric emptying was measured before the start of the experiment and at day 7 and 14 after feeding the enriched diet. After 14 days mice were anesthetized and blood was collected by cardiac puncture. The stomach and hypothalamus were removed. The stomach was cut open along the greater curvature. A strip was cut vertically from the top to the bottom of the central part of the stomach for mRNA preparation while the right part was used for protein extraction.

### Experimental Design: Oral Administration of MEDICA 16 and Grifolic Acid

Overnight fasted (15 h) WT mice were gavaged with either 150 µl 1.8% DMSO in H_2_O (vehicle) or 150 µl MEDICA 16 (10 nmol/kg) (Santa Cruz Biotechnology) or grifolic acid (10 nmol/kg) (Wuxi Chemicals) in 1,8% DMSO. Mice were habituated to gavage administration before the start of the experiment. Mice were sacrificed 40 min after gavage and blood was collected by cardiac puncture. The stomach was removed and rinsed.

### Radioimmunoassay (RIA) for Ghrelin

Blood samples (EDTA (2 mg/ml) and aprotinin (500 kIU/ml)) were centrifuged and acidified (10%) with 1 N HCl. Samples were extracted on a Sep-Pak C18 cartridge (Waters Corporation), vacuum-dried and subjected to ghrelin RIA.

The stomach was boiled and homogenized in 3 volumes of water with protease inhibitors (MP Biomedicals) and 9 volumes of 6% acetic acid. After boiling, the homogenate was centrifuged. The supernatant was removed, diluted and subjected to RIA.

The RIA was performed as previously described [27] with ^125^I[Tyr^24^] human ghrelin [1]–[23] as tracer and with an in house developed rabbit antibody raised against human ghrelin [14]–[28] (Ab2066, final dilution 1∶3000), which recognizes both octanoylated and desoctanoylated ghrelin. For the determination of octanoylated ghrelin, a rabbit antibody against human ghrelin [1]–[8] was used (Ab5004, final dilution 1∶100.000) which does not cross react with desoctanoyl ghrelin.

### Quantitative Real-Time PCR (qPCR)

Total RNA was extracted from the stomach with the RNeasy Mini kit (Qiagen) and subjected to DNAase treatment. Total RNA was isolated from the hypothalamus with the Trizol reagent and samples were purified with a RNA purification kit (Roche Diagnostics). The RNA was reverse transcribed to cDNA using Superscript II Reverse Transcriptase (Invitrogen). The Q-PCR reaction was run on a Lightcycler® 480 system (Roche Diagnostics) using LightCycler® 480 SYBR Green I Master mix. The following primers were used: Agouti-related peptide (AgRP): forward gCggAggTgCTAgATCCA, reverse AggACTCgTgCAgCCTTA; ghrelin: forward CCAgAggACAgAggACAAgC, reverse ACATCgAAgggAgCATTgAA; GOAT: forward ATTTgTgAAgggAAggTggAg, reverse CAggAgAgCAgggAAAAAgAg; GPR120: forward gTCCCATCATCATCACCATC, reverse gAT ggCCAgATgACCAggTC. Relative expression levels of all samples were calculated with the LightCycler® 480 software and were expressed relative to GAPDH and corrected for inter-run variability. Expression of GAPDH was stable in the different experimental conditions.

### Breath Test for Gastric Emptying

Gastric emptying in WT, gust^−/−^ and GHS-R^−/−^ mice fed a glyceryl trioctanoate-enriched diet was measured with a non-invasive ^13^C octanoic acid breath test as previously described [32]. The gastric half excretion time (T_half_) was calculated from the ^13^CO_2_ excretion curves [33].

### Immunohistochemistry

Stomach tissues were fixed with 4% paraformaldehyde for 2 h (4°C) followed by cryoprotection in 25% sucrose at 4°C overnight. For the staining in the ghrelinoma cell line, cells were seeded on a cover slip and after an overnight growth, fixed with 4% paraformaldehyde for 30 min at room temperature. Sections (12 µm) or cells were first incubated for 2 h in 0.1 M PBS containing 10% donkey serum, 0.3% Triton X-100 and then incubated with goat anti-GPR40 (Santa Cruz Biotechnology) or rabbit anti-GPR120 (MBL International Corporation) at 4°C overnight. After washing, the sections were incubated with donkey anti-goat or anti-rabbit Alexa594 (Santa Cruz Biotechnology) for 2 h a room temperature. For the immunofluorescence double staining experiments, sections were subsequently incubated with rabbit anti-α-gustducin, rabbit anti-α-transducin, goat anti-ghrelin all from Santa Cruz Biotechnology, rabbit anti-ghrelin (Ab2066) or rabbit anti-octanoyl ghrelin (Ab5044). These primary antibodies were visualized using donkey anti-goat or anti-rabbit Alexa488 (Santa Cruz Biotechnology) as secondary antibody. Sections were mounted with vectashield hard set medium (Vector Laboratories) and visualized under a fluorescence microscope (Olympus BX41).

### Cell Culture

MGN3-1 cells [34] were cultured in DMEM supplemented with 10% fetal bovine serum and 1% penicillin (100 u/ml) and streptomycin (100 µg/ml) at 37°C in 5% CO_2_. MGN3-1 cells were seeded at 6×10^6^ cells/well and cultured for 24 h in 12-well plates. Cells were incubated for 4 h with vehicle (Hepes (vehicle for octanoic acid and α-linolenic acid), 0.1% or 0.2% DMSO (vehicle for MEDICA16 and grifolic acid)) or increasing concentrations (10^−6^–10^−4^ M) of the indicated reagents (octanoic acid (Merck), α-linolenic acid (Sigma-Aldrich), MEDICA16 or grifolic acid) [19], [34], [35] at 37°C in 5% CO_2_ before collecting the medium. The media were acidified (10%), applied to Sep-Pak C18 cartridges and processed for RIA as described above.

### Small-interfering RNA Experiments

A pool of four small-interfering RNA (siRNA) 21-mers targeting the sequence of GPR120 was purchased from Dharmacon Research. To detect off-target effects, a mixture of four siRNA constructs (Non-Targeting siRNA Pool, Dharmacon Research) was used as a negative control siRNA. Transfection of siRNA (25 nM) was accomplished with Interferin (Polyplus transfection). After 48 hours, cells were stimulated for 4 hours with vehicle, octanoic acid (10^−4^ M) or α-linolenic acid (10^−5^ M) and the culture medium was processed as described above. GPR120 mRNA expression levels were determined by qPCR.

### Statistical Analysis

Results are presented as means ± SEM. Data (body weight, gastric emptying) obtained from measurements performed at different time points in the same mice were analyzed with Repeated measures ANOVA analysis. All other data were analyzed with One or Two Way ANOVA analysis (factor: diet and genotype). In case of significant factor effects, tests with contrasts were performed to locate pairs of factor levels with significant differences in the examined variables. Data were analysed with Statistica 10.0 (StatSoft) and significance was accepted at the *P*<0.05 level.

## Results

### Effect of a Glyceryl Trioctanoate-enriched Diet on Octanoyl Ghrelin Production: Role of α-gustducin

**Figure 1:**
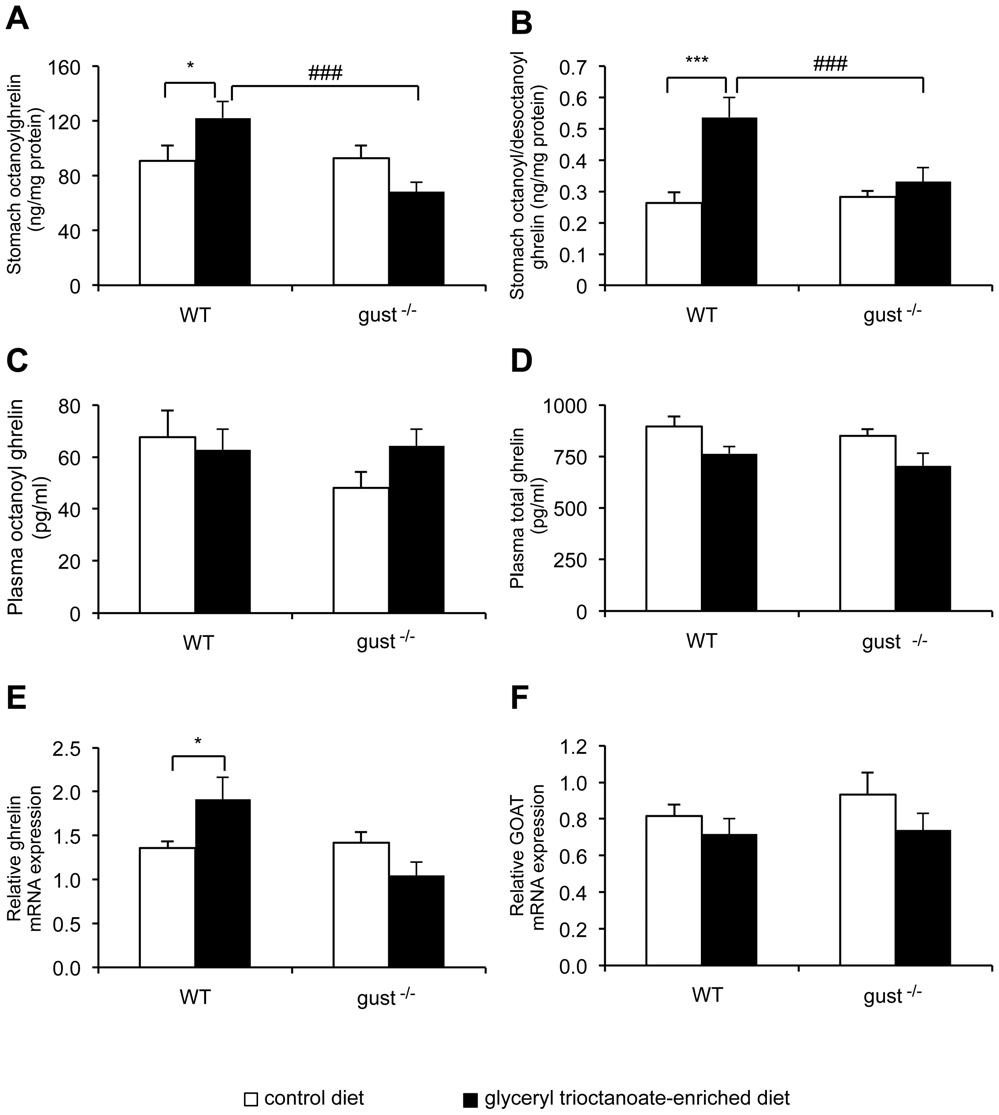
Role of α-gustducin in the effect of a glyceryl trioctanoate-enriched diet (OD) on ghrelin levels. (*A*) Octanoyl ghrelin levels in stomach extracts from WT (n = 8–15) and gust^−/−^ (n = 8–13) mice fed a control diet (CD) (white bars) or OD (black bars) for two weeks. (*B*) Octanoyl to desoctanoyl ghrelin ratio in stomach extracts of WT and gust^−/−^ mice. (*C*) Octanoyl ghrelin and (*D*) total ghrelin levels in plasma of WT (n = 16–20) and gust^−/−^ (n = 17–19) mice fed a CD or OD for two weeks. (*E*) Ghrelin mRNA and (*F*) GOAT mRNA expression in stomach of WT (n = 13–17) and gust^−/−^ (n = 14–18) mice fed a CD or OD for two weeks. All values are means ± SEM’s. *: *P*<0.05, ***: *P*<0.0005 CD vs. OD, ###: *P*<0.001 WT vs. gust^−/−^.

To examine the role of the gustatory G-protein, α-gustducin, on the octanoyl modification of ghrelin, ghrelin was extracted from the stomach of WT and gust^−/−^ mice, given *ad libitum* access to a glyceryl trioctanoate-enriched diet (OD) or a control diet (CD) during two weeks. Two way ANOVA analysis indicated a significant influence of genotype × diet (*P*<0.01). The octanoyl ghrelin concentrations were significantly increased (*P*<0.05) in the WT mice but not in the gust^−/−^ mice (Figure 1A). Total ghrelin levels were not changed by the OD in WT mice (CD: 411±15 vs. OD: 364±28 ng/mg protein) but reduced (*P*<0.001) in gust^−/−^ (CD: 418±20 vs. OD: 264±21 ng/mg protein). The octanoyl to desoctanoyl ghrelin ratio was significantly (*P*<0.0005) increased in the wild type mice but not in the gust^−/−^ mice (Figure 1B). Plasma octanoyl and total ghrelin levels remained unaffected by the glyceryl trioctanoate-enriched acid diet in both genotypes (Figure 1C and D).

Feeding mice an OD for 2 weeks increased ghrelin mRNA expression in WT mice but not in gust^−/−^ mice (Figure 1E). Two way ANOVA Analysis indicated a significant influence of genotype × diet (*P*<0.005). GOAT mRNA expression levels were not altered by the OD in both genotypes (Figure 1F). For all parameters investigated ghrelin levels did not differ under control conditions between WT and gust^−/−^ mice.

### Physiological Consequences of Feeding Mice a Glyceryl Trioctanoate-enriched Diet

**Figure 2:**
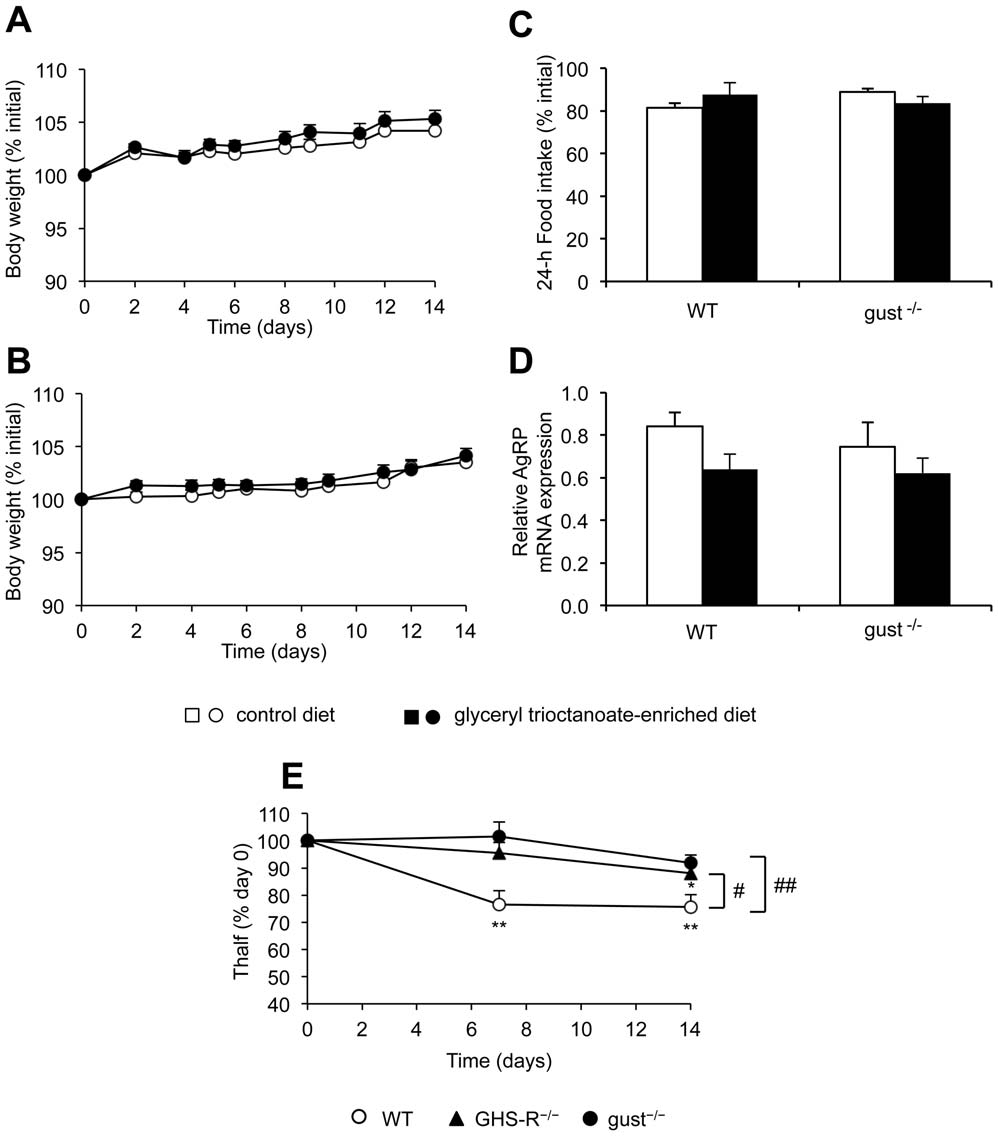
Role of α-gustducin in the effect of a diet enriched with glyceryl trioctanoate on body weight, food intake, hypothalamic AgRP mRNA expression and gastric emptying. (*A, B*) Time-dependent changes in body weight of WT (*A*) or gust^−/−^ (*B*) mice fed a CD (open symbols) or OD (filled symbols) (n = 18 mice per group) for two weeks. Results are expressed as a % of body weight before the start of the experiments. (*C*) 24-h food intake measured in WT and gust^−/−^ at day 14 on a CD or OD (n = 8 mice per group). Results are expressed as % of 24-h food intake measured before the start of the experiment. (*D*) Hypothalamic AgRP mRNA levels in WT or gust^−/−^ mice after two weeks on CD or OD (n = 10–14 mice per group). (*E*) Gastric emptying measured before the start of the experiment and at day 7 and 14 after feeding an OD in WT (open circles), gust^−/−^ (filled circles) or GHS-R^−/−^ (filled triangles) (n = 8 per genotype). All values are means ± SEM’s and are expressed as a % of their respective T_half_ value at day 0 when mice were on the control diet. *: *P*<0.05, **: *P*<0.001 vs. day 0, #: *P*<0.05 WT vs. GHS-R^−/−^, ##: *P*<0.01 WT vs. gust^−/−^.

Both food intake and body weight were measured during two weeks in WT and gust^−/−^ fed a control diet or an octanoate-enriched diet. Repeated measures ANOVA analysis showed no effect of the diet on body weight in both genotypes (Figure 2A and B). At day 14 no differences in food intake were observed between the two diets in both genotypes (Figure 2C). Consistent with the lack of effect on food intake, Two way ANOVA analysis indicated no significant changes on the mRNA expression of the orexigenic neuropeptide AgRP, mediating the effect of ghrelin on food intake in the brain, in both genotypes (Figure 2D).

Gastric emptying was measured before and during the two weeks the mice were fed an octanoic-enriched diet (figure 2E). Gastric half excretion time (T_half_) did not differ between the genotypes (T_half_: WT: 139±14 min, gust^−/−^: 120±4 min, GHS-R^−/−^: 134±13 min) at the start of the experiment when mice were on the control diet. After feeding a glyceryl trioctanoate-enriched diet, repeated measures ANOVA analysis revealed a significant influence of genotype × diet (P<0.01) with significant differences in gastric half excretion time between WT and both GHS-R^−/−^ (*P*<0.05) and gust^−/−^ mice (*P*<0.01). Planned comparisons showed that T_half_ was significantly (*P*<0.001) accelerated at day 7 (23.5±5.1%) and day 14 (24.4±5.0%) in WT mice and at day 14 (11.9±3.4%, *P*<0.05) in GHS-R^−/−^ mice. In gust^−/−^ mice gastric emptying was not influenced by the diet.

### The Role of Fatty Acid Receptors GPR40 and GPR120 in Fatty Acid Sensing of the Ghrelin Cell

#### Localization of GPR40 and GPR120 in the gut

**Figure 3:**
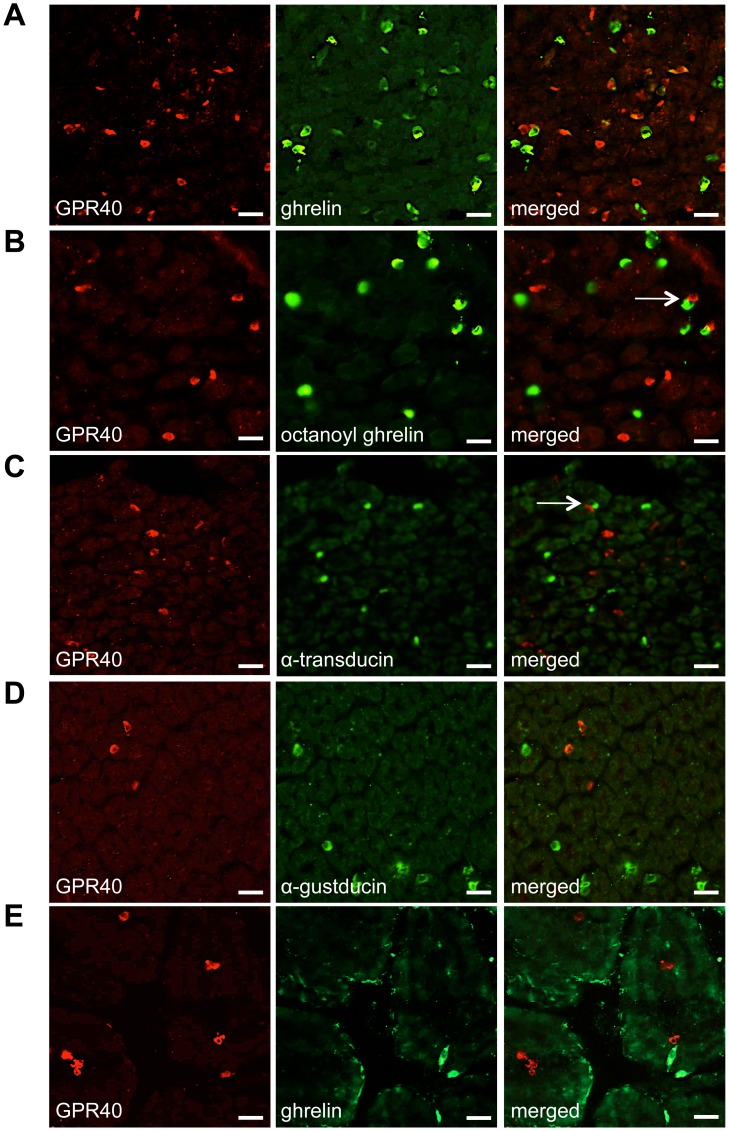
Immunofluorescence colocalization studies between GPR40 and ghrelin or the gustatory G-proteins in sections of the mouse stomach and duodenum. (*A*) Double-immunofluorescence staining showing colocalization between anti-GPR40 staining (red) and anti-total ghrelin staining (green) in endocrine cells. (*B*) GPR40 (red) immunoreactive endocrine cells did not colocalize with octanoyl ghrelin (green) immunoreactive endocrine cells, but some GPR40 positive cells were in close proximity with octanoyl ghrelin positive cells as pointed by the arrow. (*C*) No colocalization of GPR40 (red) and α-transducin (green) in stomach endocrine cells. (*D*) Double staining of GPR40 (red) and α-gustducin (green) in endocrine cells. (*E*) Double staining between GPR40 (red) and total ghrelin (green) in mouse duodenum. No colocalization is detected. Bar  = 25 µm.

Immunofluorescence staining revealed the presence of GPR40 immunoreactive cells in sections of the corpus of WT mice. Several (64±6%) GPR40 positive cells colocalized with ghrelin (Figure 3A). This ghrelin antibody did not discriminate between octanoyl and desoctanoyl ghrelin. Colocalization studies with an antibody specific for octanoyl ghrelin revealed that GPR40 did not colocalize with octanoyl ghrelin containing cells, although some cells were in close contact with each other (indicated by an arrow in Figure 3B). The gustatory G-proteins, α-gustducin and α-transducin, are present in octanoyl containing ghrelin cells [27]. GPR40 did not co-localize with α-transducin but we observed cells that were in close contact (indicated by an arrow in Figure 3C). Also α-gustducin did not show costaining with GPR40 positive cells (Figure 3D). In the duodenum, ghrelin cells were less abundant, and no colocalization between GPR40 and desoctanoyl ghrelin or octanoyl ghrelin cells could be observed (Figure 3E).

**Figure 4:**
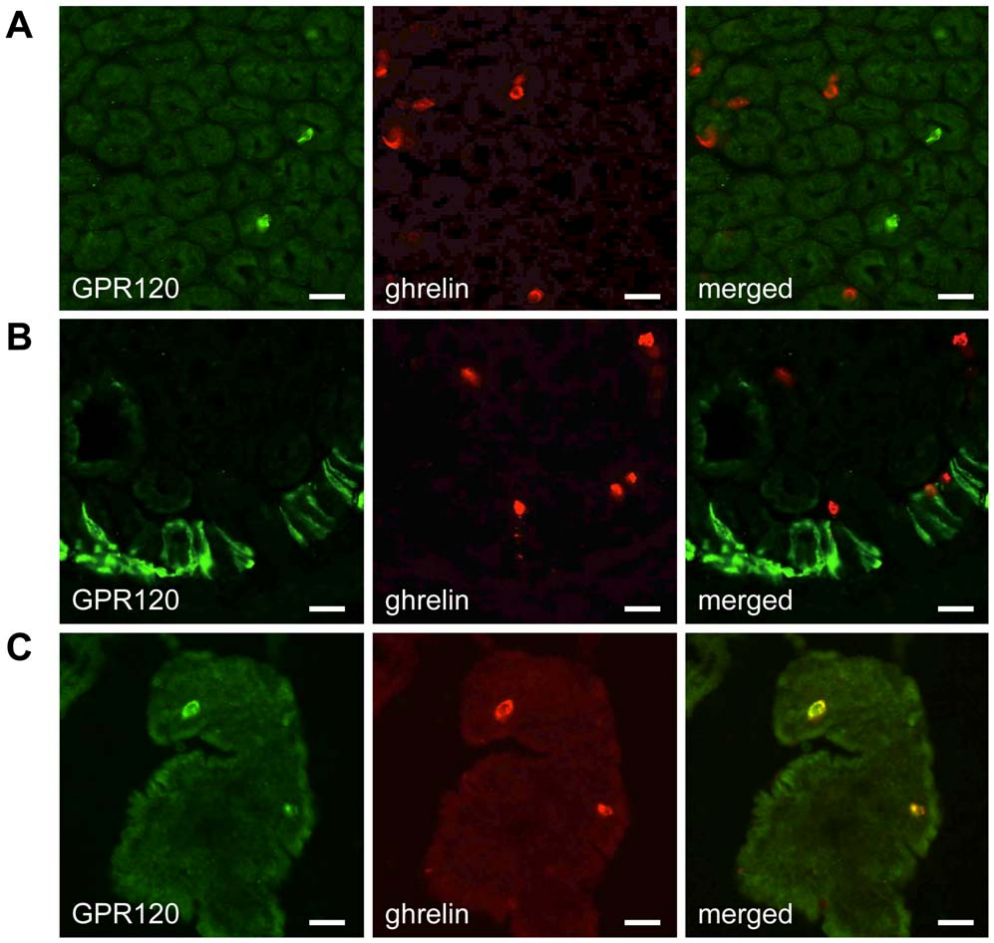
Immunofluorescence colocalization studies between GPR120 and ghrelin in sections of the mouse stomach and duodenum. (*A*) Double immunofluorescence staining showing no colocalization between anti-GPR120 (green) and anti-total ghrelin (red) staining in endocrine cells of the stomach. (*B*) GPR120 (green) immunoreactive staining was present in the brush cells at the limiting ridge. No colocalization was observed with ghrelin positive cells, but some were in close proximity. (*C*) GPR120 (red) positive cells did colocalize with total ghrelin (green) in sections of the mouse duodenum. Bar = 25 µm.

The presence of GPR120 was shown in endocrine cells (Figure 4A) and in brush cells (Figure 4B) at the limiting ridge of the mouse stomach. GPR120 immunoreactive cells did not colocalize with ghrelin (Figure 4A and B). GPR120 positive cells were also observed in the duodenum. In contrast to the stomach, these cells colocalized for 81±10% with the ghrelin cell population (Figure 4C). Unfortunately, we could not perform colocalization studies between GPR120 and octanoyl ghrelin or the gustatory G-proteins, α-gustducin and α-transducin, because the antibodies were from the same host species.

#### Role of GPPR40 and GPR120 in the effect of medium and long chain fatty acids on ghrelin secretion in the ghrelinoma cell line

**Figure 5:**
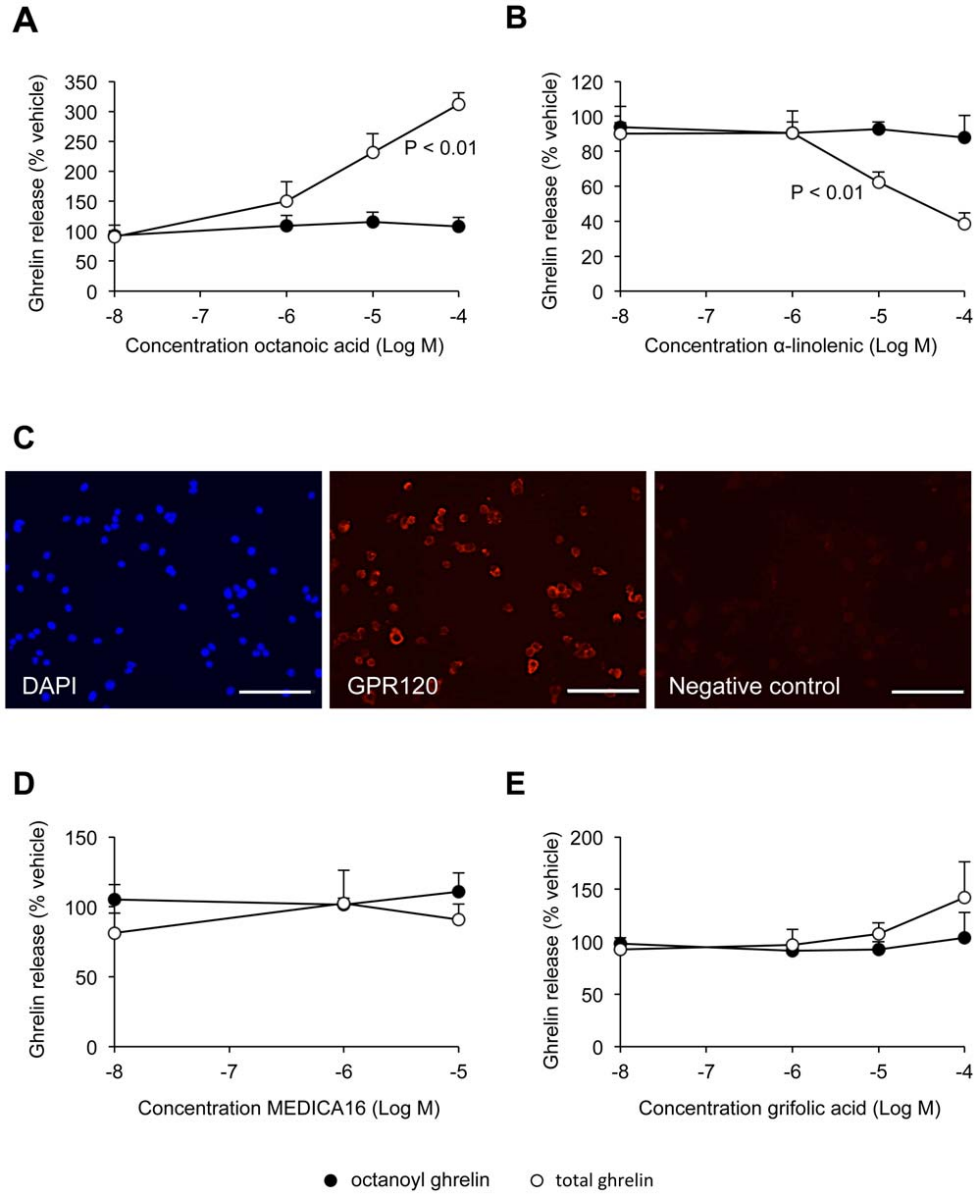
Effect of FFA stimulation on ghrelin secretion in the ghrelinoma cell line. MGN3-1 cells were stimulated with different concentrations of octanoic acid (*A*), α-linolenic acid (*B*), MEDICA16 (*D*) or grifolic acid (*E*). Both octanoyl (filled circles) and total ghrelin (open circles) levels were measured in the medium 4 h after administration of the FFA. (*C*) Immunofluorescence staining for DAPI (left), GPR120 (middle) and negative control (right) in the ghrelinoma cell line. Bar  = 100 µm. All values are means ± SEM’s of 4 independent experiments performed in triplicate and are expressed as a % of ghrelin secretion obtained after stimulation with vehicle.

The effect of a glyceryl trioctanoate-enriched diet on the octanoylation of ghrelin was mimicked *in vitro* by addition of octanoic acid to the culture medium of the ghrelinoma cell line, MGN3-1. Octanoic acid significantly (One way ANOVA analysis, *P*<0.01) increased octanoyl ghrelin secretion in a concentration-dependent manner (10^−8^ M: 90±19%, 10^−6^ M: 150±33%, 10^−5^ M: 231±31%, 10^−4^ M: 312±19%). In contrast, total ghrelin levels were not affected by administration of octanoic acid (Figure 5A).

Next we examined the effect of the long chain fatty acid, α-linolenic acid, on ghrelin secretion. Stimulation of the MGN3-1 cells with α-linolenic acid resulted in a concentration-dependent decrease (One way ANOVA analysis, *P*<0.01) in octanoyl ghrelin levels compared to vehicle stimulated cells. In contrast total ghrelin levels were not affected (Figure 5B).

GPR120 mRNA but not GPR40 mRNA is expressed in the ghrelinoma cell line. The expression of GPR120 at the protein levels was confirmed by immunofluorescence staining against GPR120 (Figure 5C). To investigate a possible role of GPR40 and GPR120 in the fatty acid sensing mechanisms of the ghrelin cell, we administered the selective agonists MEDICA16 and grifolic acid to the ghrelinoma cell line, respectively. As expected stimulation with MEDICA16 did not affect ghrelin levels since GPR40 is absent in the MGN3-1 cells (Figure 5D). However also application of the GPR120 agonist, grifolic acid, was without any effect (Figure 5E).

**Figure 6:**
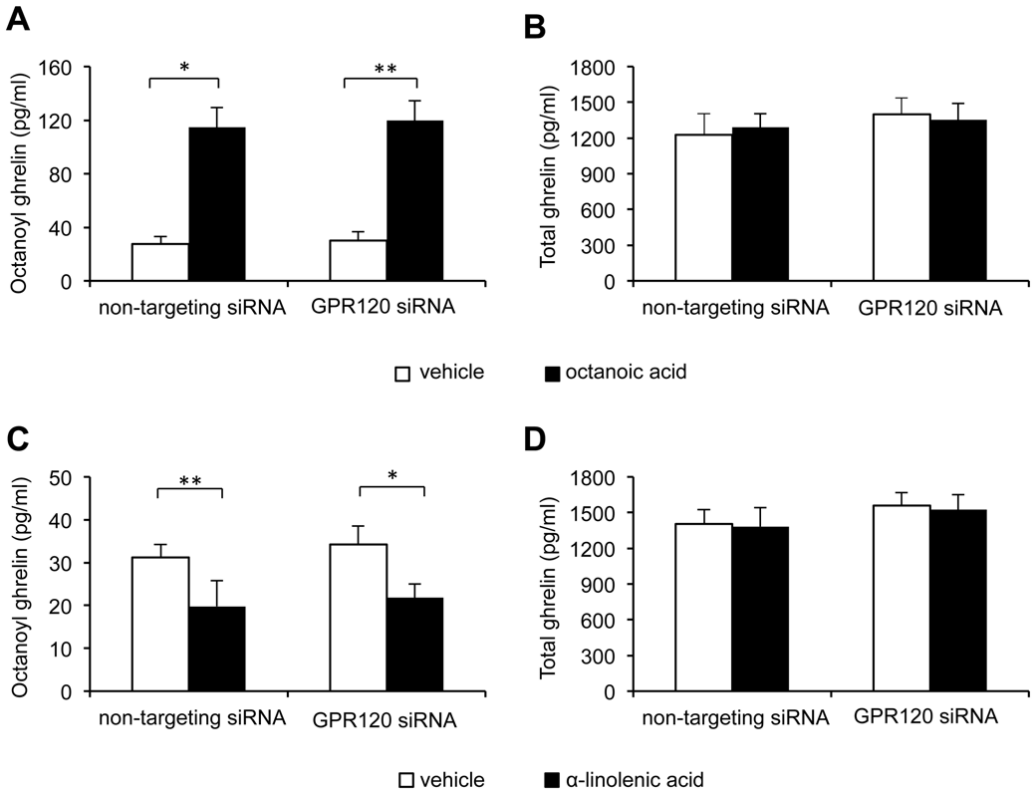
Role of GPR120 in the effect of octanoic acid (*A–B*) and α-linolenic acid (*C–D*) on ghrelin levels in the ghrelinoma cell line. Octanoyl and total ghrelin secretion was measured 4 h after administration of vehicle, octanoic acid (10^−4^ M), or α-linolenic acid (10^−5^ M) to MGN3-1 cells transfected with GPR120 siRNA or a non-targeting siRNA pool as negative control. All values are means ± SEM’s of 4 independent experiments performed in sevenfold. *: *P*<0.05, **: *P*<0.01 vehicle vs. octanoic acid or α-linolenic acid.

To determine whether the FFA receptor GPR120 was involved in the effects of octanoic acid or α-linolenic acid on octanoyl ghrelin secretion, we transfected MGN3-1 cells with siRNA for GPR120. The transfection resulted in 50±4% knockdown of GPR120 mRNA expression (4 independent experiments in triplicate). Application of octanoic acid (10^−4^ M) to GPR120 siRNA transfected cells increased octanoyl ghrelin levels (vehicle: 30±6 pg/ml vs. octanoic acid: 120±15 pg/ml) (Figure 6A) to the same extent as cells transfected with the non-targeting siRNA (vehicle: 28±6 pg/ml vs. octanoic acid: 115±21 pg/ml). Total ghrelin levels remained unaffected in both GPR120 siRNA and non-targeting siRNA transfected cells (Figure 6B). Similarly, the inhibitory effect of the long-chain fatty acid, α-linolenic acid (10^−5^ M) on octanoyl ghrelin levels was not blocked by transfection of MGN3-1 cells with GPR120 siRNA (Figure 6C). Total ghrelin levels were not affected in both GPR120 siRNA and non-target siRNA transfected cells after stimulation with α-linolenic acid (Figure 6D). Therefore we could not assess a function to GPR120 in the ghrelinoma cell line.

#### Effect of the GPPR40 agonist, MEDICA16, and the GPR120, grifolic acid, on ghrelin secretion *in vivo*

**Figure 7:**
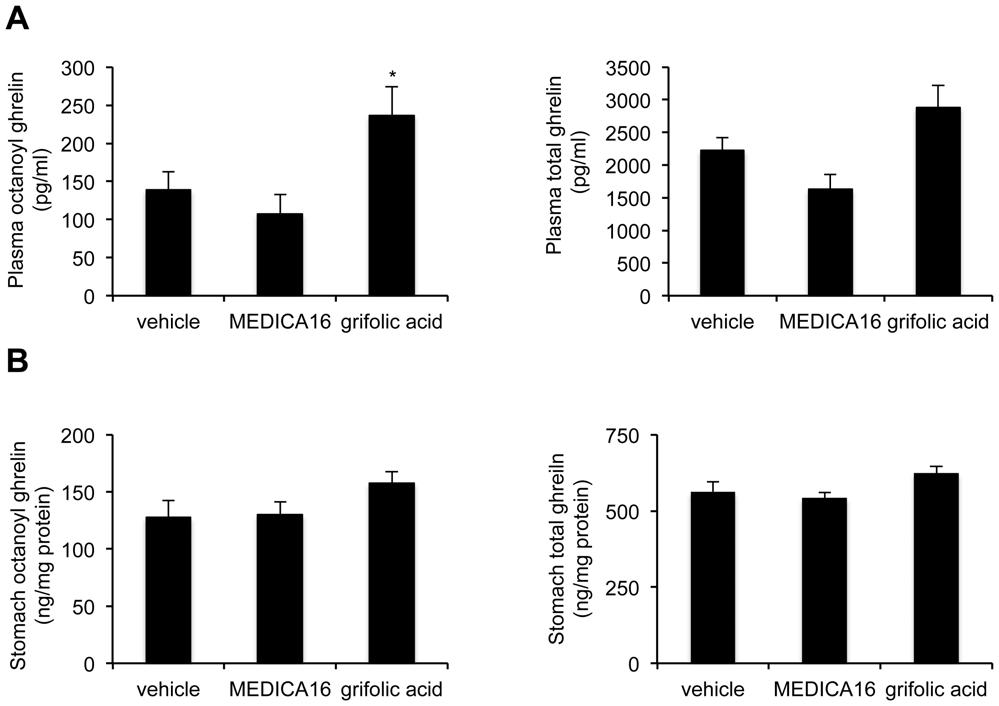
Effect of MEDICA16 and grifolic acid on ghrelin secretion *in vivo*. Octanoyl ghrelin (left) and total ghrelin (right) levels were measured in plasma (*A*) or in stomach extracts (*B*) 40 min after gavage of 0,18% DMSO (vehicle), 10 nmol/kg MEDICA16 or grifolic acid. All values are means ± SEM’s of experiments performed in 8 mice. *: *P*<0.05 vs. vehicle.

The effect of intragastric administration of 10 nmol/kg MEDICA16 or grifolic acid on ghrelin secretion was investigated. One way ANOVA analysis indicated a significant (P<0.05) effect of treatment. MEDICA 16 did neither affect plasma octanoyl nor total ghrelin levels. In contrast, after gavage of grifolic acid, plasma octanoyl ghrelin levels were increased 1.7–fold (P<0.05) (Figure 7A). A similar trend was observed for the effect on plasma total levels (P = 0.08). The ratio of octanoyl versus desoctanoyl ghrelin levels was not affected (vehicle: 0.073±0.011, MEDICA16∶0.077±0.016, grifolic acid: 0.080±0.011). Stomach ghrelin content was not affected by intragastric administration of the GPR40 or GPR120 agonist (Figure 7B).

## Discussion

Recent data stress the role of lipid sensing mechanisms in the regulation of energy balance [36], [37], [38]. Also the ghrelin cell must sense lipids since the secretion of ghrelin is inhibited by lipids and ghrelin octanoylation is modified by dietary lipids, in particularly by the availability of MCFA [11], [12], [17], [39]. The function of ghrelin may therefore not exclusively be that of a hunger signal reflecting an empty stomach, but the ghrelin-GOAT system may act as an energy-sensor to alert the central nervous system about the presence of a calorie-rich environment to optimize lipid storage and permit growth [39]. However, the signaling pathways involved in sensing of lipids by the ghrelin cell are not known. In the current paper we described for the first time a role for the gustatory G-protein, α-gustducin, in the octanoylation of ghrelin. Feeding mice a diet enriched with glyceryl trioctanoate for two weeks increased octanoyl ghrelin levels in stomach extracts from WT but not from gust^−/−^ mice. We have previously shown that α-gustducin is present in the brush cells of the stomach in close contact with octanoyl containing ghrelin cells but also in endocrine cells immunoreactive for octanoyl ghrelin [27]. Since orally ingested medium chain triglycerides can passively diffuse from the GI tract to the portal system, it is therefore questionable if the α-gustducin-containing brush cells, which are in direct contact with the lumen, are involved in the sensing of these lipids. Our data also show that the octanoylation of ghrelin can be increased *in vitro* by addition of octanoic acid to the culture medium of the ghrelinoma cell line, MGN3-1. However, in contrast to the *in vivo* situation, the increased octanoyl ghrelin was also effectively secreted in the cell culture medium. This reinforces the hypothesis that the α-gustducin containing endocrine ghrelin cells contain the machinery to sense the octanoic acid directly, probably from signals coming via the blood stream and thus independent from luminal stimuli transmitted via the brush cells.

In contrast to octanoyl ghrelin levels, total ghrelin levels in the stomach were not affected by the diet, insinuating that only the octanoylation process was influenced by an excessive availability of octanoic acid in the diet. Similar findings were obtained by addition of octanoic acid to the cell culture medium of the ghrelinoma cell line. Furthermore the glyceryl trioctanoate-enriched diet did not affect GOAT expression, although a change in the activity of the enzyme cannot be excluded. Also the octanoyl ghrelin levels in the blood were not altered by the diet implying that ghrelin octanoylation and the secretion of octanoyl ghrelin probably represent two independent processes. This may be a defense system of the body to prevent enhanced signaling of ghrelin under conditions of positive energy balance provided by the excess of lipids. Gahete *et al.* [40] already showed that GOAT expression in the stomach is a good predictor of octanoyl ghrelin in the plasma. Ghrelin levels did not differ between WT and gust^−/−^ mice receiving a control diet suggesting that α-gustducin is less important in sensing other octanoyl species than glyceryl trioctanoate as well as other non-8-carbon species which have also been found to be incorporated in the ghrelin peptide [17].

The increased octanoyl ghrelin levels in the stomach resulted in an acceleration of gastric emptying in WT mice, but not in gust^−/−^ mice. The effect on emptying was also significantly different between WT and GHS-R^−/−^ mice, suggesting that the effect on gastric emptying is mediated via the increase in octanoyl ghrelin levels which involves α-gustducin. The local increase in ghrelin in the stomach may affect gastric contractility via a direct interaction with peripheral ghrelin receptors present on enteric nerves [33], [41] or via activation of vagal afferent endings [42]. It remains to be investigated whether an octanoic-acid enriched diet may be useful to treat hypomotility disorders in patients.

Several fatty acid responsive proteins have been identified that may play a role in initiating fatty acid transduction including fatty acid binding protein CD36 [43] and several GPCRs including GPR40 and GPR120 [18], [19]. The immunofluorescence stainings showed that 64% of GPR40 immunoreactive endocrine cells colocalized with ghrelin in the mouse stomach but not in the duodenum. We have previously shown that the mouse stomach contains two ghrelin cell populations: cells containing octanoyl and desoctanoyl ghrelin and cells staining for desoctanoyl ghrelin only [27]. We showed that GPR40 only colocalizes with the desoctanoyl containing ghrelin population. Nevertheless, some octanoyl containing ghrelin cells were in close contact with GPR40 positive desoctanoyl ghrelin containing cells. GPR40 was not colocalized with the gustatory G-protein α-gustducin, but some GPR40 immunoreactive cells were in close contact with α-transducin. These findings together with the observation that GPR40 is not colocalized with octanoyl ghrelin cells neither in the stomach nor in the duodenum suggest that it is rather unlikely that this receptor is directly involved in the octanoylation process of ghrelin. Secondly we revealed the expression of GPR120 in both the brush and endocrine cells in the mouse stomach. Some GPR120 containing brush cells were in close contact with ghrelin immunoreactive cells. In contrast to GPR40, GPR120 positive endocrine cells colocalized with ghrelin in the duodenum but not in the stomach.

A role for GPR120 in the octanoylation of ghrelin was studied *in vitro* using the mouse ghrelinoma cell line, MGN3-1. The protein expression of GPR120 in the MGN3-1 cells was validated by immunofluorescence studies. Transfection of the cells with siRNA specific for GPR120 did not block the increase in octanoylation induced by addition of octanoic acid to the cell culture medium. Unfortunately we could not perform siRNA studies for GPR40 since we could not validate the presence of GPR40 in this cell line.

Sensing of fatty acids may not only affect the octanoylation of ghrelin but may also be important in the regulation of ghrelin secretion. To further investigate the fatty acid sensing mechanisms of the ghrelin cell we studied the effect of the long chain fatty acid, α-linolenic acid, on ghrelin secretion. We showed for the first time that α-linolenic acid specifically inhibits the release of octanoyl ghrelin but not of total ghrelin. These findings imply that the release of octanoyl and desoctanoyl ghrelin may be regulated differently. Our data suggest that the ghrelin endocrine cell contains the machinery to sense MCFA and LCFA directly.

The GPR120-containing brush cells in the stomach or the GPR120-containing “open type” ghrelin cells in the duodenum make GPR120 a plausible candidate for sensing long-chain fatty acids in the lumen. Previous studies already demonstrated a role for GPR120 in α-linolenic acid-induced GLP-1 and CCK secretion in STC-1 cells [21], [22], [44]. Transfection of MGN3-1 cells with siRNA for GPR120 decreased GPR120 mRNA expression but did not block α-linolenic acid-induced octanoyl ghrelin suppression. In accordance, a specific agonist for GPR120, grifolic acid [35], was not able to mimic the effect observed with α-linolenic acid on ghrelin release. In accordance with the absence of GPR40 in the ghrelinoma cell line, no effect on ghrelin secretion was observed with the GPR40 agonist, MEDICA16 [35]. However, intragastric administration of grifolic acid increased plasma ghrelin secretion *in vivo*. Since the ghrelin content in the stomach was not affected by gavage of grifolic acid it is likely that ghrelin was secreted from the ghrelin containing cells in the duodenum which are colocalized with GPR120. Since grifolic acid also stimulates GLP-1 secretion from the enteroendocrine cell line, STC-1, indirect effects mediated via the release of GLP-1 or other gut hormones cannot be excluded [35]. Further studies are warranted to investigate this.

The full characterization of the receptors and transporters, as well as the signaling pathways that mediate fatty acid detection within the GI tract is of major relevance due to their apparent contribution to important functions, like ghrelin octanoylation and secretion and thus energy intake. We reported for the first time a role for the G-protein, α-gustducin, in the octanoylation process of ghrelin and provide evidence that the ghrelin cell can sense MCFA (octanoic acid) and LCFA (α-linolenic acid) directly with opposite effects on octanoyl ghrelin secretion. Our *in vitro* and *in vivo* data suggest that GPR40 is not of major importance in the fatty acid sensing cascade of the ghrelin cell, but *in vivo* studies with grifolic acid point towards a direct or indirect role of GPR120 in ghrelin secretion. The role of the membrane lipid-binding protein CD36 which plays a major role in the orosensory perception of LCFAs in the mouse also warrants further investigation [45]. Studies in CD36 null mice support an important role for duodenal CD36 fatty acid translocase in the dietary uptake of oleic acid [37].

Recent findings suggest that the expression of gustatory-signaling elements, including GPR120 and α-gustducin, is increased in morbidly obese patients [46]. Since these chemosensory cells are part of the complex mechanisms regulating energy homeostasis, this opens doors for strategies aimed at interfering with lipid sensing mechanisms in the gut for the treatment of obesity and other eating disorders.
